# Effect of ethylenediamine tetraacetic acid (EDTA) and maleic acid as root-end conditioners on the sealing ability of ProRoot mineral trioxide aggregate (MTA) and Biodentine as root-end filling materials: An in vitro study

**DOI:** 10.34172/joddd.025.41232

**Published:** 2025-03-31

**Authors:** Sumaiya Zafar, Yawar Ali Abidi, Samira Adnan, Maham Muneeb Lone, Farah Afroz Khan, Isma Sajjad

**Affiliations:** ^1^Department of Operative Dentistry and Endodontics, Faculty of Dentistry, Sindh Institute of Oral Health Sciences, Jinnah Sindh Medical University, Karachi, Pakistan

**Keywords:** Biodentine, EDTA, Maleic acid, MTA

## Abstract

**Background.:**

Apicoectomy is often required to treat a non-healing endodontic lesion. Materials used during this procedure, such as root-end conditioners and retrograde filling materials, can significantly affect the success. Mineral trioxide aggregate (MTA) has been used as a root-end filling material for a long time; however, success has also been reported with some novel materials such as Biodentine. The present study compared the sealing ability of MTA and Biodentine in root-end cavities following apicoectomy after using EDTA and maleic acid as root-end conditioners.

**Methods.:**

The apical one-third of 60 extracted teeth were resected, followed by root-end preparation of 3 mm. The samples were divided into 4 groups of 15 each. The roots in groups 1 and 2 were conditioned with EDTA and maleic acid in groups 3 and 4. Retrograde cavities in groups 1 and 3 were filled with MTA and in groups 2 and 4 with Biodentine. Microleakage was checked using the dye penetration method. One-way ANOVA and post hoc Tukey tests were used for statistical analyses.

**Results.:**

Group 1 showed the highest (2.94±0.112), while group 4 had the least dye penetration (2.55±0.213).

**Conclusion.:**

It can be concluded that Biodentine showed better sealing ability after using maleic acid than MTA and can be used as an alternative to MTA in root-end surgeries.

## Introduction

 The primary aim of root canal treatment is to clean, shape, and fill the root canal system three-dimensionally and form a coronoapical barrier against the penetration of microbiota and its byproducts.^1,2^ Although conventional root canal treatment is effective in most cases, failure does occur sometimes. In these instances, non-surgical re-treatment is employed. However, when all the efforts of an orthograde endodontic therapy fail, apicoectomy is a viable treatment option.^3^

 Apicoectomy is a common surgical endodontic procedure that involves the extirpation of the apical portion of the tooth along with the adherent diseased soft tissues.^4^ The procedure includes periapical curettage, root-end resection, root-end preparation, and root-end filling. The cementum covering the apical dentin is usually exposed during this procedure. To allow the regeneration of periodontium and to create a successive apical seal, filling the canals with only gutta-percha is not adequate. Root-end preparation and filling are recommended to enhance a good apical seal after the root-end resection.^5^

 Several materials have been used for root-end filling, such as intermediate restorative material (IRM), amalgam, super ethoxy benzoic acid (Super-EBA), glass ionomer cement (GIC), composite resin, mineral trioxide aggregate (MTA), and Biodentine, among others. However, none of the materials have been shown to possess all the ideal properties.^6^ MTA has been most successful clinically compared to other root-end filling materials since it is less cytotoxic, biocompatible, and prevents microleakage.^7^ However, it does have its disadvantages, such as prolonged setting time, cost, difficult handling, potential discoloration, and lower flexural and compressive strengths.^8^ Biodentine is a relatively new bioactive material introduced as a dentin substitute. It is formulated using MTA-based cement. As a root-end filling material, it has shown superior apical sealing and regeneration of the periodontium compared to MTA due to its improved physical properties, such as faster setting time, better handling, and increased strength, despite the latter’s established clinical success and biocompatibility.^5^ The manufacturer of Biodentine claim that it is superior to MTA.^9^

 Various root-end conditioners are used to remove the smear layer to enhance mechanical adhesion and cellular activity in terms of growth and development. EDTA, commonly used in a concentration of 17% to remove the smear layer, is one of the common root end conditioners. Due to its neutral pH, it is also biocompatible with the periradicular tissues.^10^

 Maleic acid is a mild organic acid, which is also biocompatible and is capable of removing the smear layer; however, it is not used extensively in periapical surgeries.^11^

 A study by Villamayor et al^12^ showed the bond strength of Biodentine to be 16.48 ± 3.92 MP and that of MTA to be 15.67 ± 3.07 MPa when used as root-end filling materials.

 The present study evaluated the effectiveness of Biodentine compared to MTA as a root-end filling material in terms of apical seal quality after treating the root end with two conditioners: EDTA and maleic acid.

###  Hypothesis

 There is a difference in the sealing ability of Biodentine and ProRoot mineral trioxide aggregate.

## Methods

 After exemption was obtained from the institutes’ ethical review committee (#JSMU/IRB/2019/267), this study was conducted at the Department of Operative Dentistry, Sindh Institute of Oral Health Sciences, Jinnah Sindh Medical University for two months. A sample of 60 single-rooted extracted teeth with fully formed apices was used in this in vitro experimental study. The sample size was calculated using an open Epi sample size calculator (Open Source Epidemiologic Statistics for Public Health, Version. https://www.openepi.com).

 A study by Mohamed Nabeel et al.^5^ found that the mean leakage value of ProRoot MTA as a root-end filling material was 0.80 ± 0.63 after one day, and that of Biodentine was 0.20 ± 0.42. Keeping these values as a reference and power of t-test (1-β) at an 80% level and significance (α) at 5%, the estimated sample size was n = 13 in each group. However, the sample size was increased to accommodate any procedural errors.

 Decoronation was performed using a cutting disc: Yeti magic disk (Yeti Dental Produkte, GmbH, Germany). Endodontic access was gained for each tooth and a #10 K-file (SybornEndo, Mexico) was used to determine the working length 1 mm short of the apex. Each canal was prepared using rotary ProTaper universal files (DENTSPLY Maillefer, Ballaigues, Switzerland) up to F2. The irrigation protocol involved the use of 2 mL of 3% sodium hypochlorite (Endosol, Pakistan) and 5 mL of 17% EDTA (META BIOMED CO. LTD, Germany) solution, followed by a final flush with 5 mL of 3% sodium hypochlorite using a 5-mL syringe with a 23-gauge needle (Star Plus, China). Using lateral compaction technique, gutta-percha (Dentsply, Sirona) and Sealapex (Kerr Dental, USA) were used to obturate the root canals, and the quality of obturation was assessed radiographically. GIC (GC Gold, Tokyo, Japan) was used to seal all canal orifices after obturation. All the samples were then stored at 37 ± 1 °C and 100% relative humidity for 7 days. The root end of each tooth was resected perpendicularly to the long axis of the root in the apical third with a cross-cut fissure bur (Mani, Japan). A retrograde cavity of 3 mm was prepared using a straight fissure diamond bur (Mani, Japan).

 The teeth were then divided into 4 groups of 15 each. The cavities in groups 1 and 2 were irrigated with 5 mL of 17% EDTA and groups 3 and 4 with 5 mL of 7% maleic acid and then dried. All irrigating solutions were introduced into the root canals using a 5-mL syringe with a 23-G needle (Star Plus, China). The total application time for the final irrigation solutions was 1 minute. Root-end cavities in groups 1 and 3 were filled with MTA (Angelus Soluções Odontológicas, Londrina, Brazil) and in groups 2 and 4 with Biodentine (Septodont, U.S.A) using MTA carrier (Dovgan, USA). To ensure the complete setting of root-end filling material, the samples were placed in an incubator at 37 °C for 48 hours with a wet gauze covering the roots and then coated with three layers of nail varnish except for the resected end and allowed to dry. Apical leakage was evaluated using the methylene blue dye penetration technique. The apical half of each root was submerged in 2% methylene blue dye (Vista-Blue solution) for 24 hours at 37 °C and 100% humidity. The roots were then removed, and excess dye material was removed by rinsing it under tap water for 15 minutes and then air-dried. Nail varnish was removed using a scalpel, and all the specimens were mounted into transparent acrylic resin to allow vertical sectioning of root in a buccolingual direction using a metal cutting disc under copious irrigation. The specimens were then photographed, and the images were transferred to Adobe Photoshop CC 2018 to measure the depth of dye penetration along the interface of the root-end filling and the root canal wall.

## Results

 In each group, 15 readings were taken. Table 1 shows the mean depths of dye penetration in the four experimental groups. Group 1 (EDTA and MTA) showed the highest, while group 4 (Maleic acid and Biodentine) exhibited the least dye penetration at the interface of root-end filling material and root canal wall. One-way ANOVA and post hoc Tukey tests were applied to determine any significant difference in leakage between the four groups, which was statistically significant (Table 2).

**Table 1:** Comparison of mean dye penetration depths in millimeters (mm) in the experimental groups

**Experimental groups**	**N**	**Mean±SD (mm)**
I (EDTA and MTA)	15	2.94 ± 0.112
II (EDTA and Biodentine)	15	2.79 ± 0.213
III (Maleic acid and MTA)	15	2.60 ± 0.178
IV (Maleic acid and Biodentine)	15	2.55 ± 0.213

N: number of teeth in each group

**Table 2:** The difference in dye penetration at the root end between the 4 experimental groups

**Group 1**	**Groups 2, 3, and 4**	**Mean±SD**	***P*** value*
EDTA + MTA	EDTA + Biodentine	0.1533 ± 0.067	0.118
	Maleic acid + MTA	0.266 ± 0.067	0.001*
	Maleic acid + Biodentine	0.393 ± 0.067	0.000*

*One-way ANOVA was applied. *P* value was set at 0.05 (*P <*0.05). Post hoc Tukey tests were applied.

## Discussion

 One of the main objectives of conventional root canal treatment is to form a three-dimensional

 coronoapical hermitic seal between the periodontium and root canal system. Postoperative diseases can be managed by either non-surgical or surgical approaches. Novel methods and materials used during periradicular surgery have significantly enhanced the success rate. During periapical surgery, cementum-bounded apical dentine is exposed; hence, a root-end filling material is required for a good apical seal. The type of root-end filling material used significantly affects the treatment outcome.

 This study was conducted to determine the root-end conditioner that would result in the most favorable outcome following root-end filling using MTA and Biodentine without adversely affecting the sealing properties of these materials. Following root-end resection, the resected surface is conditioned using different agents. The purpose is to decontaminate the root surface, remove the smear layer, create an environment conducive to cementogenesis, and enhance periradicular healing. Several methods have been used to this end, broadly divided into physical and chemical.

 Commonly used chemicals include citric acid, phosphoric acid, tetracycline hydrochloride, EDTA, and maleic acid, among others.^13,14^ EDTA and maleic acid were used in this research. When using these conditioners alongside the root end filling materials, their effect must be determined in terms of enhancing or deteriorating the sealing ability of the root end filling. EDTA has been used for root-end conditioning for a long time and has been effective in removing the smear layer. However, it has a few drawbacks, such as a reduction in dentin microhardness, reduced efficacy in the apical third, and cytotoxicity. Maleic acid is a mild organic acid with less cytotoxicity than EDTA.^15^ In a study by Kuruvilla et al 7% maleic acid was more effective in removing the smear layer than EDTA, especially in the apical third.^16,17^ Both peritubular and intertubular dentin erode when radicular dentin is exposed to 17% EDTA for longer than one minute. Maleic acid’s mild acidic nature and ability to remove the smear layer, especially in the apical third, makes it an excellent agent as an alternative to other root-end conditioners.^18^

 A root-end filling material should have certain attributes to be considered an ideal material, such as biocompatibility, sealing ability, dimensional stability, marginal adaptation, bioactivity, and antimicrobial properties. Several materials have been used as root-end filling materials; however, the ones tested in this study included MTA and Biodentine, and their sealing ability was determined using methylene blue dye penetration. Dye penetration is one of the most common, non-toxic, and inexpensive methods of detecting microleakage in vitro; hence, it was used in our study.^19^

 The results of this study showed that Biodentine, when used in combination with maleic acid, had the least amount of leakage (2.55 ± 0.213), followed by MTA with maleic acid (2.60 ± 0.178), Biodentine with EDTA (2.79 ± 0.213), and MTA with EDTA (2.94 ± 0.112) in descending order. This could be due to the ability of maleic acid to remove the smear layer and demineralize intertubular dentin more effectively than EDTA. One of the reasons for this is the higher acidity of maleic acid, having a pH value of 1.05. In addition, the root-end filling material Biodentine has been shown to have better sealing and mechanical properties than MTA, less porosity and pore volume in the set material, fast setting, and formation of tag-like structures in dentine, which could be the reason for less dye penetration and subsequent leakage in the present study.^19^

 A study to compare the sealing ability of Biodentine and MTA as root-end filling material using two different preparation techniques (bur vs. ultrasonic) concluded that less microleakage was seen in Biodentine and ultrasonic preparation than in MTA and bur preparation.^19^

 Another study evaluated the marginal adaptation of three root-end filling materials: GIC, MTA, and Biodentine. Good marginal adaptation and lowest marginal gaps were observed in Biodentine, followed by MTA, and highest in GIC.^20^ A stereomicroscopic study conducted to compare the marginal seal between MTA, GIC, and Biodentine as root-end filling material concluded that although all materials showed microleakage, it was the least in Biodentine.^21^

 One of the strengths of this study was the use of maleic acid, which has not been widely analyzed and used in apicoectomy procedures. According to the results of our study, maleic acid did not adversely affect the properties of root-end filling materials.

 Some limitations include a small sample size. Burs instead of ultrasonic tips generated more debris and smear layer,^22^ which could have affected the outcome of this study. Dye penetration can have its drawbacks, such as the small size of dye molecules compared to bacteria, which makes it difficult to determine if the prevention of microleakage of the dye would actually depict that microorganisms would also be prevented from percolating into the root canal.^23^

 Careful use of ultrasonic devices for root-end preparation instead of burs can generate less debris and smear layer, enhancing the sealing abilities of root-end filling materials. Long-term in vivo studies should be used to determine the effect of blood contamination on the sealing ability of Biodentine and the nature and longevity of the bond obtained from the dentine‒Biodentine interface.

## Conclusion

 Within the limitations of this in vitro study, it can be concluded that the use of maleic acid in

 combination with Biodentine provides an excellent apical seal with minimum leakage; hence, it can be used as an alternative to MTA.

## Competing Interests

 None.

## Ethical Approval

 CODE: 267(#JSMU/IRB/2019/267), Jinnah Sindh Medical University, Sindh Institute of Oral Health Sciences.
